# Modeling residual cholesteatoma growth rate: a meta-analysis of individual participant data

**DOI:** 10.1007/s00405-026-10326-5

**Published:** 2026-06-01

**Authors:** Borbála Körmendy, Judit Kálmán, Kata Illés, Péter Fehérvári, Alexander Schulze Wenning, Rita Nagy, Peter Hegyi, Francois Simon, Tamás Horváth

**Affiliations:** 1 Centre for Translational Medicine, Semmelweis University, Budapest, Hungary; 2 Department of Otorhinolaryngology, Head and Neck Surgery, Bajcsy-Zsilinkszky Hospital, Maglódi út 89-91, Budapest, 1106 Hungary; 3 University of Veterinary Medicine Budapest, Budapest, Hungary; 4 Heim Pál National Pediatric Institute, Budapest, Hungary; 5 Institute of Pancreatic Diseases, Semmelweis University, Budapest, Hungary; 6 Hôpital Necker-Enfants Malades, Université Paris Cité, AP-HP, Paris, France

**Keywords:** Cholesteatoma, Residual, Growth rate, DWI-MRI

## Abstract

**Purpose:**

To create a model of residual cholesteatoma growth rate that can be used to estimate optimal timing of follow-up imaging.

**Methods:**

Following the recommendations of the PRISMA-IPD guideline and after registering our study protocol on PROSPERO (CRD42023465320), a systematic search was completed in September 2023 and updated in July 2024, conducted in PubMed, Web of Science, Embase, and Cochrane Library. Reference lists of the resulting articles were also searched for potential inclusions. Our search yielded 11,274 articles. Studies with patients of any age and sex diagnosed with residual cholesteatoma were included if data on cholesteatoma size at follow-up were available, collected from imaging results or through second-stage surgery. For statistical analysis, a Monte Carlo simulation approach was applied, and a baseline random-intercept mixed-effects model was fitted.

**Results:**

We included 11 articles reporting individual data from 163 patients. All but one of these articles proved to be high risk of bias. Our simulation models suggest that 95% of cholesteatomas reach or grow past the size of 3 millimetres (threshold for imaging) after 28–35 months and 95% of reach or grow past the size of 5 millimetres (threshold for intraoperative identification) after 47–58 months.

**Conclusion:**

Analyzing previously published data suggests that residual cholesteatoma may only be radiologically detectable on non-EPI DWI MRI after 28–35 months postoperatively in 95% of patients, indicating delayed follow-up for average risk patients compared to current clinical practice. However, this simulation model, based on retrospective data, aims to uncover growth patterns rather than posing as a new guideline.

**Supplementary Information:**

The online version contains supplementary material available at https://doi.org/10.1007/s00405-026-10326-5.

## Introduction

Although many advances were made in cholesteatoma surgery, the risk of residual disease may still be as high as 40% [1–4]. Thus, follow-up after surgery is indispensable. The traditional way of monitoring residual disease used to be second-look surgery performed 1 year after the first procedure [5, 6] even in cases without apparent signs of cholesteatoma during otomicroscopic examination. The emergence of non-echoplanar diffusion weighted magnetic resonance imaging (non-EPI DWI MRI) techniques has changed the obvious need for surgical surveillance, as this imaging modality shows high sensitivity and specificity for cholesteatoma [7, 8]. It has been proven to be more cost-effective compared to the planned second-look surgery [9], however, there is still no established guideline for the optimal timing of radiological examinations [10].

There are different aspects that should be considered when timing the follow-up targeted imaging or the second-stage surgery. If follow-up imaging is performed too early, residual cholesteatoma might go unnoticed due to its small size [11, 12]. If imaging is delayed, residual disease holds the risk of complications or may grow to an extensive size requiring a more radical approach for removal, which may negatively affect postoperative quality of life [13]. Knowing the growth rate of residual cholesteatoma could help determine the ideal time point to detect cholesteatomas in most patients without risking the development of extensive disease in the meantime.

In this meta-analysis of individual participant data, we aimed to summarize all measurements available in the literature on residual cholesteatoma growth and to create a simulation model to estimate the optimal time for follow-up [14].

## Methods

This growth and residual cholesteatoma detectability model with systematic review was based on the recommendations of the Preferred Reporting Items for Systematic Review and Meta-Analyses of Individual Participant Data (PRISMA-IPD) guideline [15] and the Cochrane Handbook [16]. The study protocol has been registered on PROSPERO (CRD42023465320) and was fully adhered to except for one adjustment with the exclusion criteria, as our search yielded only one eligible article containing congenital cholesteatoma growth data. As their different etiopathogenesis may also be associated with disparate growth dynamics from acquired cholesteatomas, we ultimately decided to exclude congenital cholesteatomas from our study.

### Inclusion and exclusion criteria

**Fig. 1:**
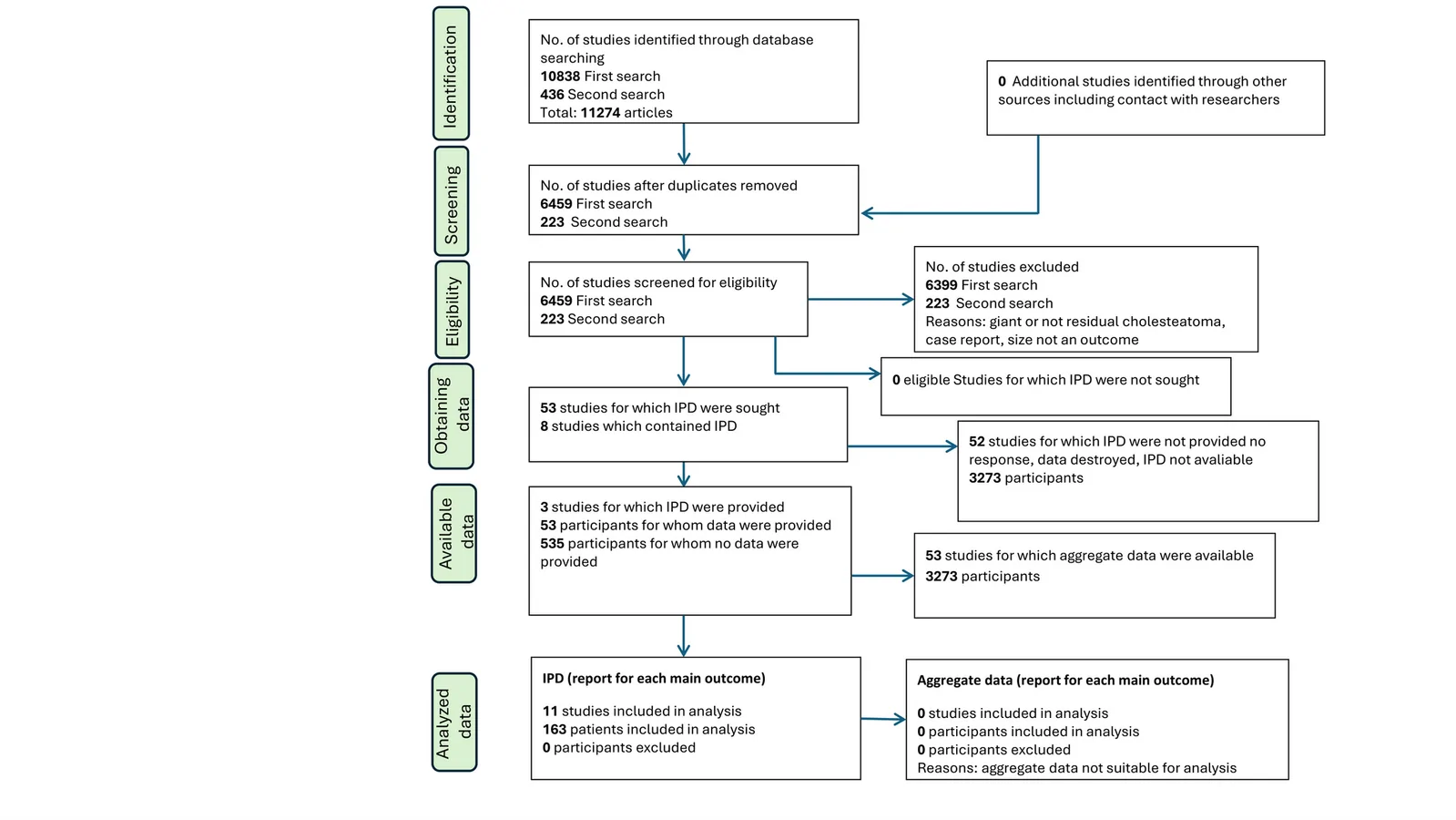
PRSMA-IPD flowchart of study selection process. IPD: individual participant data, No: number

Patients of any age and sex diagnosed with cholesteatoma were included, if data on cholesteatoma growth were available, collected from imaging results or through direct visualization in the operating room. Although our primary goal was to include both acquired and congenital cholesteatoma, the latter was excluded after the selection process for the reasons indicated above. Published prospective or retrospective cohort studies and observational studies written in English were included, whereas reviews, conference abstracts, case reports, guidelines and animal studies were excluded. We also excluded studies where residual cholesteatoma was detected within one month after surgery, because the calculation of growth rate would be extremely distorted with such a short follow-up time. (Fig. 1.)

### Information sources and search strategy

The first systematic search was conducted on September 20, 2023, and updated on July 12, 2024 (during the data selection process, we became aware of new studies that were published containing data that might be useful for our analysis) in PubMed, Web of Science, Embase, and Cochrane Library using the search key below, with no filters:

Cholesteatoma AND (growth or follow* or follow-up or recurrence or MRI).

References included in the eligible studies were also scanned using CitationChaser [17]. Corresponding authors of further records that did not contain individual participant data on cholesteatoma size or time interval between surgery and scan were contacted via email. (Fig. 1)

### Selection process

We used a citation manager program (EndNote 20, Clarivate Analytics, Philadelphia, PA, USA) for automatic and manual duplicate removal, then title, abstract and, full text selection was performed by two reviewers (BK and JK) working independently, using Rayyan [18]. After each selection step, disagreements were resolved by a third reviewer and inter-rater reliability was assessed using Cohen’s kappa [19].

### Data collection process

Data from the eligible articles (name of the first author, year of publication, basic demographic characteristics (percentage of females, age, number of patients), age at surgery, cholesteatoma type, (if recurrent, time since last surgery), method of size measurement or imaging modality, size, if surgery technique available and location of cholesteatoma) was entered into a predefined Excel worksheet (Microsoft Corporation, Redmond, Washington, United States). Authors of articles that contained aggregate data on cholesteatoma growth were contacted, and if individual participant data were still available, we asked for anonymized data including patient age, sex, type of surgery, time elapsed between surgery and imaging, as well as cholesteatoma size. Through datasharing requests, we received combined, anonymized individual participant data (IPD) (time between surgery and imaging, cholesteatoma size) for 52 cholesteatomas from 3 articles [11, 20, 21] by the same working group at the Department of Paediatric Otolaryngology, Université Paris Cité. (Table 1.) Radiological and non-radiological methods of measurements were summarized in Online Resource 2 and Online Resource 3.

**Table 1:** Study characteristics. OCEBM: Oxford Centre for Evidence Based Medicine, F: female, CWU BOT: canal wall up with bony obliteration technique,non-EPI DWI MRI: non-echoplanar diffusion-weighted magnetic resonance imaging, mo: month, mm2: square millimeters, * = from these studies we havereceived combined, anonymized growth data for 52 cases (52 datapoints, all indirect)

First author, year of publication (Country)	Study method (OCEBM rate of evidence)	No. of cases (female %, age, data on previous operations)	No. of cases with growth data (no. of datapoints, indirect/direct)	Measurement method	Follow-up length	Mean growth rate
Baoudouin et al., 2023 (France) [20]	Retrospective study (4)	140 (NA, 9.2 ± 3.7 years, various techniques)	*	Non-EPI DWI MRI	3.2 ± 2.2 years	Not calculated
Daoudi et al., 2023 (France) [11]	Retrospective study (4)	224 (30% F, 2–18 years)	*	Non-EPI DWI MRI	27 ± 24 months	Not calculated
Delrue et al., 2019 (Belgium) [43]	Retrospective clinical record study (4)	48 (50% F, 14–84 years, post subtotal petrosectomy)	10 (10, 10/0)	EPI and non-EPI DW MRI, long and short axis diameter in coronal view	0.5–13 years (mean 3.9)	0.54 mm^2^ /month
De Foer et al., 2008 (Netherlands) [44]	Prospective and blinded study (3)	32 (31% F, 7–71 years, post CWU mastoidectomy)	2 (2, 2/0)	Intraoperative indings	11.2–40.3 months (mean 25.2)	Not calculated
Gristwood et al., 1976 (Australia) [45]	Prospective cohort study (3)	141 (50% F, 6–83 years, various techniques)	23 (29, 24/5)	Intraoperative indings	At most 6 years	epitymanic: doubles diameter every 10 mo, mastoid: doubles diameter every 25 mo
Hellingman et al., 2019 (The Netherlands) [46]	Retrospective case series (4)	10 (70%F, 12–72 years, post subtotal petrosectomy)	10 (62, 13/49)	MRI (not specified)	8-186 months (mean 59)	25.6mm3/month
Lecler et al., 2015 (France) [47]	Prospective monocentric consecutive study (3)	22 (9%F, 4–18 years)	7 (10, 10/0)	Non-EPI DWI and contrast enhanced MRI	At least 12 months	Not calculated
Pai et al., 2019 (United Kingdom) [48]	Retrospective case review (4)	12 (NA, 4–66 years, various techniques)	34 (34, 34/0)	Non-EPI DWI MRI	3.0–42.8 (Mean 8.1 years, median 6.1)	4 mm/year (median 2, range 0–18)
Simon et al., 2024 (France) [21]	Retrospective observational study (4)	223 (30%, 8.4 ± 4.3 years, various techniques)	*	Non-EPI DWI MRI	95 months ± 26.2 (range 60–186)	Not calculated
Smith et al., 2020 (United Kingdom) [49]	Retrospective longitudinal study	17 (41%F, 10–72 years)	17 (38, 17/21)	Non-EPI DWI MRI	33 months (range 6–91)	0,6 mm/year (-0,2–19.9 mm / year)
van der Toom et al., 2022 (Belgium ) [6]	Retrospective case review	271 (39% F, post CWU BOT with mastoid obliteration)	8 (11, 8/3)	Non-EPI DWI MRI	60 months (IQR 56–62)	1,8 mm/8 months

### Data items

**Fig. 2:**
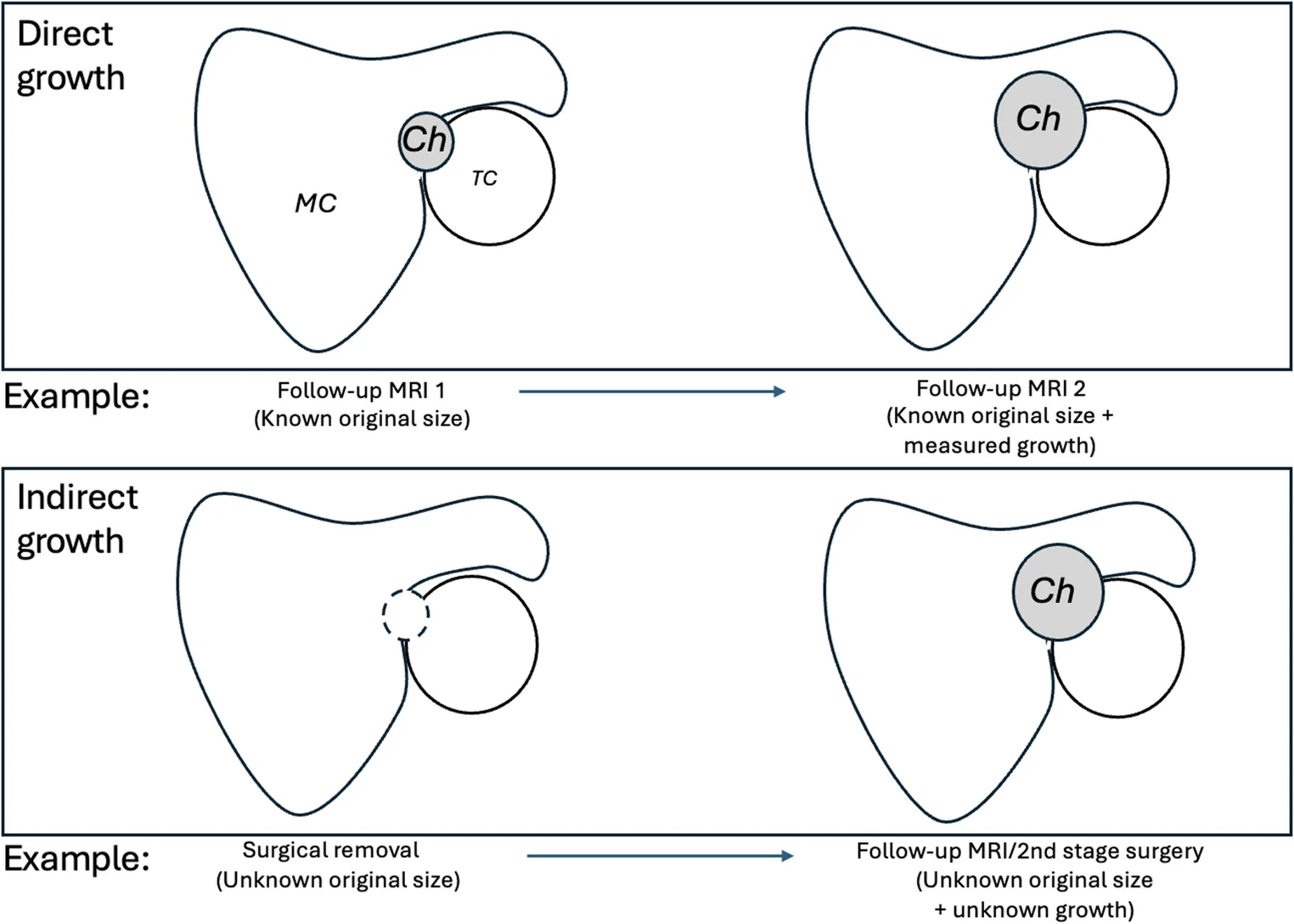
Demonstrating the distinction between indirect and direct growth data. For direct growth data, we have an exact measurement of the initial size, whereas for indirect datapoints we can make assumptions. MC: mastoid cavity, TC: tympanic cavity, Ch: cholesteatoma

Our main outcome was cholesteatoma growth rate. To calculate the growth rate, we included two types of studies. The first comprised purely non-EPI DWI MRI-based reports in which exact size measurements were available for the same residual cholesteatoma at two different time points. Patients in this group were followed, but not operated for different reasons, such as pregnancy or the SARS-CoV-2 pandemic. We termed these datapoints “direct measurements”. The second type of measurements was included in studies where, after a primary surgery, only one set of size data was available from follow-up MRI imaging or second stage. In these cases, we assumed that the initial size of the residual mass during primary surgery was below the visually detectable range; we estimated it to be zero to one millimeters in these cases, but the true size of the residuals remains unknown. These were named “indirect measurements”.(Fig. 2) In order to validate the joint calculation of the growth rate based on the direct and indirect measurements, we statistically compared the two approaches and performed further analyses to determine whether patient sex, age, and the type of surgery significantly influenced growth rate. We chose 3 mm and 5 mm thresholds to estimate the earliest time of high sensitivity and specificity imaging, and feasible intraoperative identification.

### Study risk of bias assessment

The two data reviewers assessed the risk of bias using the JBI critical Appraisal for Case Series [22] and have visualized the results using the Risk-of-bias VISualization web application [23]. Disagreements were resolved through discussion.

## Level of evidence

Level of evidence was ranked using the OCEBM 2011 Levels of Evidence Table [24] assisted by the OCEBM Background document [25].

### Statistical methods

Individual participant data were obtained from retrospective and prospective studies reporting cholesteatoma size at one or more time points. Two forms of measurements were available: direct measurements, where lesion size was recorded at two separate clinical encounters, and indirect measurements, where only follow-up size was documented. In the latter case, the clinically established assumption that cholesteatomas originate from a radiologically inapparent state of 0 mm, 0.33 mm, 0.66 mm, and 1 mm was applied to estimate growth over the observed interval, consistent with prior descriptions of early lesion morphology and radiological detectability [26–28]. Because the two measurement types represent different forms of information and may introduce systematic differences, measurement type (direct vs. indirect) was included as a fixed effect in all models, and an additional sensitivity analysis restricted to directly measured cases was performed to assess robustness, acknowledging the substantially reduced sample size. A separate simulation was also run based exclusively on radiological measurements, based on the results of the risk of bias analysis.

Lesion size measurements were extracted in three formats: two-dimensional values, from which the larger dimension was used as the effective diameter; area measurements, from which the diameter of an equivalent circle was derived; and volume measurements, from which the diameter of an equivalent sphere was computed, following established morphometric principles [28]. Monthly growth rates were calculated by dividing the change in diameter by the elapsed time in months. Because growth rates are strictly positive and typically right-skewed, the values were log-transformed to stabilize variance and improve normality, in line with standard recommendations for modeling log-normal biological processes [29, 30]. Exponentiated fixed-effect coefficients from the resulting models therefore represent multiplicative changes in the geometric mean monthly growth rate.

Linear mixed-effects models were fitted using restricted maximum likelihood estimation, with patient-specific random intercepts nested within studies to account for both within- and between-study heterogeneity [31–33]. Model fitting relied on the lme4 package, which implements well-established numerical approaches for estimating hierarchical models [34]. Covariates included sex, age at surgery, measurement type, and surgical technique, modelled separately when dictated by differences in variable availability. Confidence intervals for fixed-effect estimates were obtained using profile likelihood, a recommended approach for mixed models [34, 35]. Model assumptions were evaluated through inspection of residual distributions and fitted–residual relationships, and model fit was summarised using marginal and conditional R² values computed according to the method of Nakagawa and Schielzeth [36], as implemented in the MuMIn package [37].

To estimate the time required for cholesteatomas to reach clinically relevant sizes, a Monte Carlo simulation study was conducted. An intercept-only mixed-effects model was fitted to the log-transformed growth rates to characterise the underlying distribution of lesion expansion. The fixed-effect estimate, its standard error, and the residual variance were used to define a log-normal data-generating process, drawing on established principles of parametric bootstrapping and Monte Carlo simulation [38–40]. For each target diameter (3 mm and 5 mm), 10,000 independent Monte Carlo draws were generated by sampling log-growth rates from a normal distribution centred on the fixed-effect estimate with variance equal to the sum of its squared standard error and the residual variance. These simulated values were exponentiated and converted into expected times to reach the specified diameter through deterministic transformation. This procedure yields a distribution of plausible growth trajectories rather than replicating a specific study design, and the resulting distributions were summarised using empirical quantiles and density-based visualisations.

All analyses were conducted in R (version 4.4.2; R Core Team 2024) using the lme4 [34], lmerTest [41], and sjPlot [42] packages.

## Results

A total of 11 articles were included, encompassing 1272 patients with growth data available for 163 of them [6, 11, 20, 21, 43–49]. During all study selection steps, Cohen’s kappa was higher than 0,9. 62 (38%) were male, 32 (19,6%) female, and data on sex were not provided for 69 (42,3%) of them. Mean age was 28,3 years (range: 4–72 years). In total, 248 data points were collected from 170 indirect and 78 direct measurements. (Table 1.)

### Biological predictors of growth

**Fig. 3:**
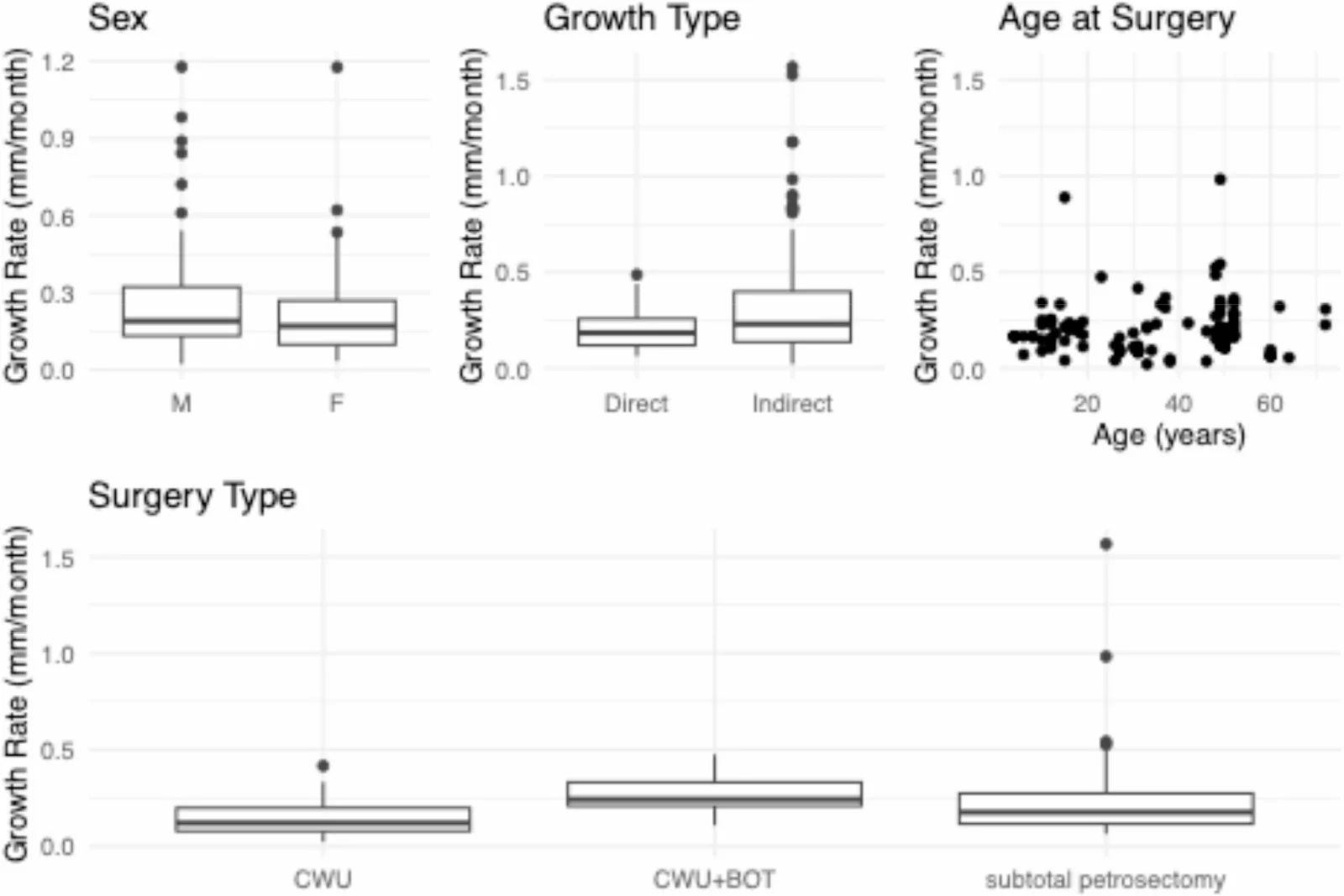
Summarization of observed relationships with the initial size of indirect cholesteatomas set to 0 mm: top row includes sex, growth type, and age; bottom row shows effects of surgical type. mm: millimetre, M: male, F: female, CWU: canal wall up, CWU + BOT: canal wall up with bony obliteration

The model, including sex and age at surgery, showed that growth rate was slightly higher in males compared to females, with an estimated 19% increase in monthly diameter growth (95% CI: 0% to 42%, *p* = 0.054). Age at surgery was not a statistically significant predictor of growth rate (1% decrease per year; 95% CI: -3% to + 1%, *p* = 0.18). The marginal R² was 0.017, and the conditional R² was 0.31, indicating that most of the variability was explained by random effects. Further analysis of age at diagnosis of residual disease was not possible due to a lack of data. (Fig. 3)

### Growth type

**Fig. 4:**
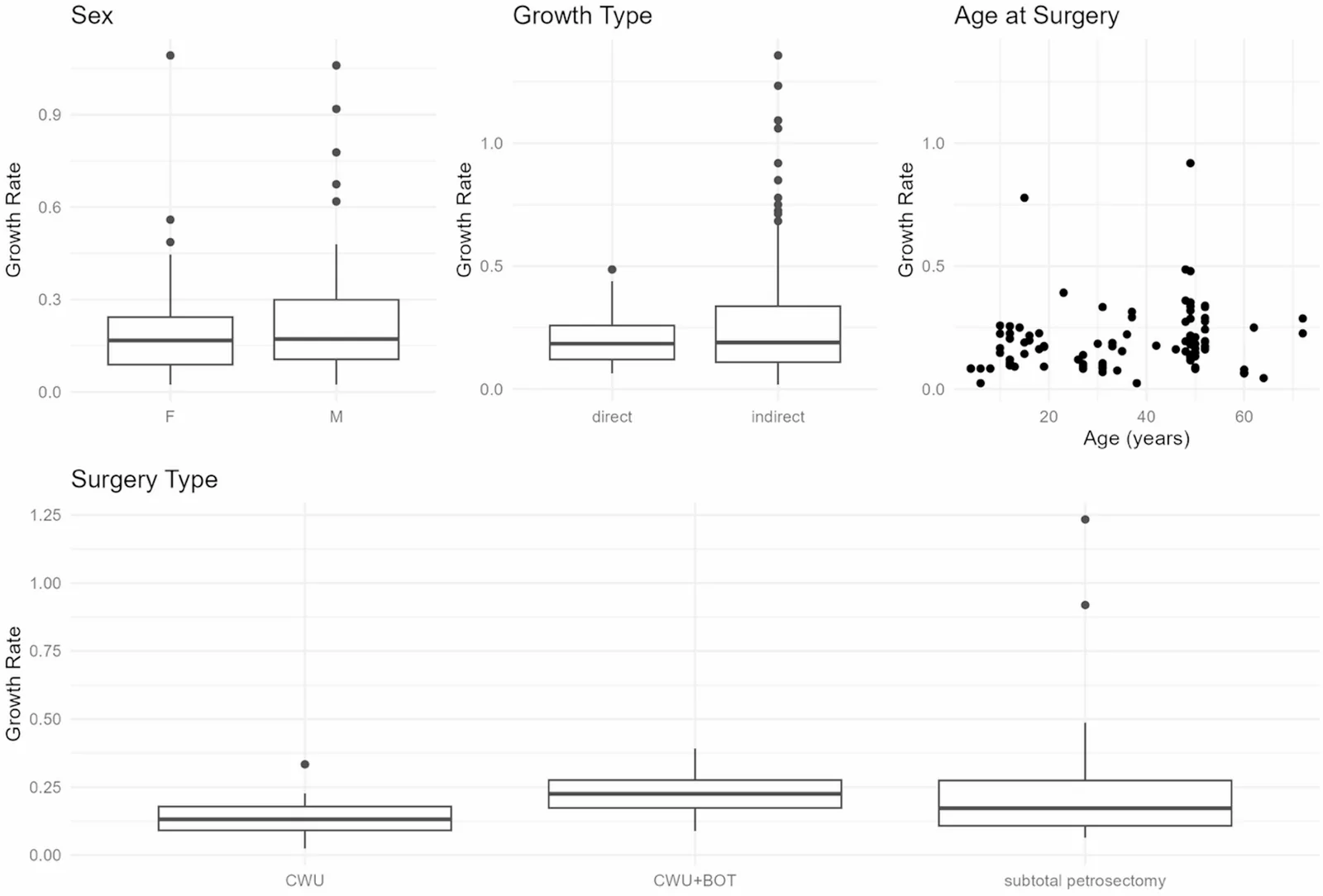
Summarization of observed relationships with the initial size of indirect cholesteatomas set to 1 mm: top row includes sex, growth type, and age; bottom row shows effects of surgical type. mm: millimetre, M: male, F: female, CWU: canal wall up, CWU + BOT: canal wall up with bony obliteration

In a separate model evaluating growth type, directly measured growth was associated with a significant, 25% lower growth rate compared to indirect measurements (95% CI: 6% to 47%, *p* = 0.01). This model had a marginal R² of 0.029 and a conditional R² of 0.28. (Fig. 3) When changing the starting point of indirectly measured growth to 1 mm, there was no significant difference between the two methods (95% CI: 6% to 47%, *p* = 0.191). This model had a marginal R² of 0.035 and a conditional R² of 0.59. (Fig. 4). Simulations with the initial size of indirect cholesteatomas set to 0.33 mm and 0.66 mm were also carried out (Online Resource Figs. 2, 3, 4 and 5.), and also with excluding non-radiologically measured datapoints (Online Resource Fig. 6–7.).

### Surgical technique

Patients who underwent canal-wall-up surgery with bony obliteration technique (CWU + BOT) had a nonsignificant, 20% higher growth rate compared to CWU alone (95% CI: -7% to + 54%, *p* = 0.14). Patients with subtotal petrosectomy (STP) had a significantly, 36% higher growth rate compared to CWU (95% CI: 6% to 75%, *p* = 0.01). Marginal R² was 0.031, and conditional R² was 0.29. (Fig. 3)

### Simulation study

**Fig. 5:**
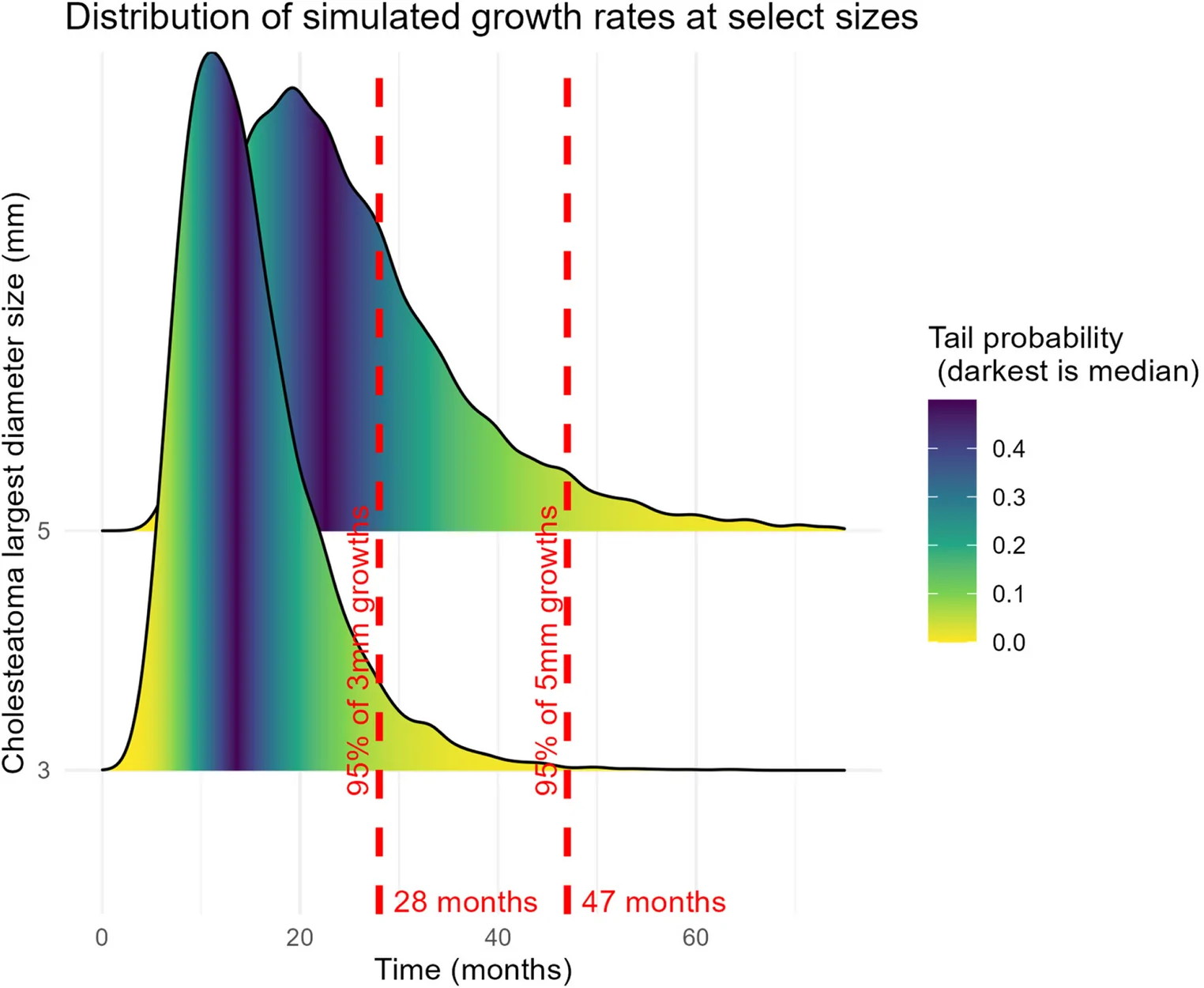
Ridge density plot of simulated time (months) required to reach 3 mm and 5 mm cholesteatoma growth targets, with the initial size of indirect cholesteatomas set to 0 mm. The figure shows the distribution of the likelihood of cholesteatomas reaching a size of 3 or 5 millimetres by a specific timepoint. Dashed red lines indicate 95th percentiles for each target

**Fig. 6:**
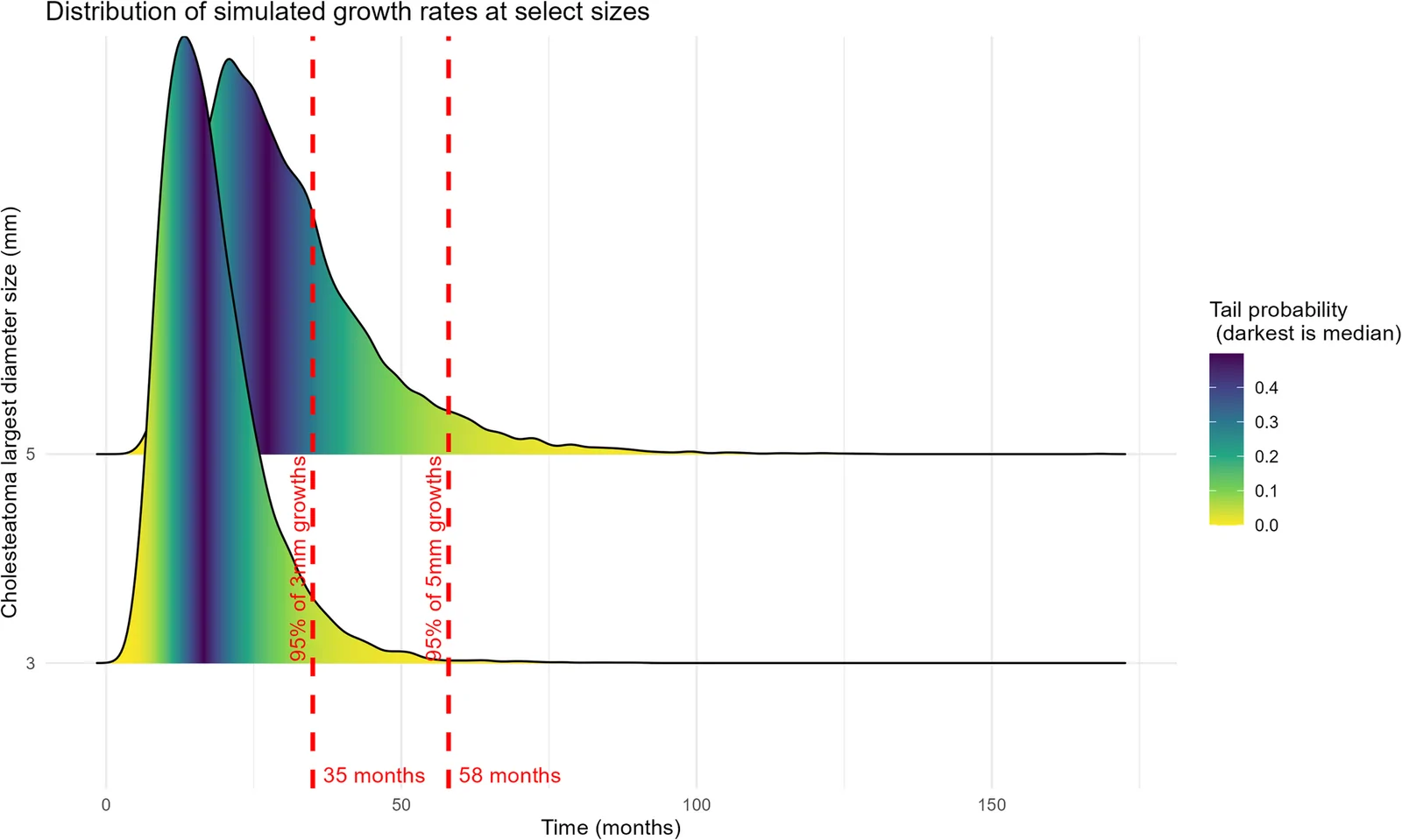
Ridge density plot of simulated time (months) required to reach 3 mm and 5 mm cholesteatoma growth targets, with the initial size of indirect cholesteatomas set to 1 mm. The figure shows the distribution of the likelihood of cholesteatomas to reach a size of 3 or 5 millimetres by a specific timepoint. Dashed red lines indicate 95th percentiles for each target

Using the baseline model with indirectly measured cholesteatomas presumed to be 0 mm as a starting point, we generated simulated growth durations to estimate the time required for cholesteatomas to reach radiological and clinical thresholds (3 mm and 5 mm). For a 3-mm target, 95% of simulated cases reached this size within 28 months. For a 5-mm target, 95% reached this size within 47 months. (Fig. 5) With a starting size of 1 mm for indirectly measured cholesteatomas, the time passed until the earliest radiological detection (3 mm) increases to 35, and to 58 months for surgical detection (5 mm). (Fig. 6) When excluding non-radiological measurements from direct cases, a 3 mm threshold was found at 27 months and 5 mm at 46 months for 95% of patients (Online Resource Fig. 6–7).

### Risk of bias assessment

As a result of the risk of bias analysis using the JBI tool for case series, all but one of our selected articles proved to be high risk of bias, most often due to heterogeneity of measurement protocols and lack of data on disease demographics. (Online Resource 1.)

## Discussion

In this review article, we introduce a growth simulation and residual disease detectability model based on cholesteatoma sizes measured during imaging or second-look procedures. These results may support future recommendations for the optimal time of follow-up MRIs, for which to our knowledge no widely accepted protocol exists yet. Recent large-scale studies regarding long-term cholesteatoma follow-up showed that a surprisingly high percentage of residual cases were only discovered several years after surgery, and prematurely performed follow-up imaging holds the risk of yielding false negative results [6, 11, 12, 21]. Our growth model which was created using all the available data including previous reports seems to be in accordance with this, since we found that 95% of cholesteatomas measuring 3 mm in diameter can be detected at 28–35 months postoperatively, while 5 mm diameter residual disease becomes visible at 47–58 months after the surgery in 95% of the patients. Although targeted MRI imaging was found to be able to detect residual cholesteatoma as small as 3 mm [50, 51], we also analyzed a 5-mm diameter as a threshold value. This size compares without relevant clinical consequences to 3 mm, but it further reduces the risk of false negative examinations [52, 53], also considering that surgical techniques such as cartilage or cortical bone columella may affect specificity [54, 55]. Following the traditional statistical approach [56], we defined the detection threshold as 95% of the patients with residual cholesteatoma with the aforementioned sizes.

Considering the results of this model, the optimal, cost-effective screening for residual cholesteatoma should be performed no earlier than 2 years in average-risk patients after the first or previous surgery. In contrast to this suggestion, there are many cases of residual disease detected as early as a few months after removal [57]. Some patients have a high risk of developing residual disease [58, 59], and they should be subjected to consistent follow-up, including early MRIs. As more data are needed for a comprehensive risk stratification system for recidivism, surgeons should individually plan follow-up for patients based on intraoperative findings. Another recurring argument against delayed follow-up is to avoid developing complications. However, it was shown during the SARS-CoV-2 pandemic that increased waiting periods for cholesteatoma surgery did not lead to higher rates of major complications [60].

Our model was based on two different measurement methods of growth found in the literature. In one group 2 or more size measurements of the same cholesteatoma were performed at two or more different timepoints. By subtracting the earlier measurement from the latter, we could calculate the exact initial size of residual growth (direct measurement). In the other group we had a single datapoint, collected from imaging results or second-look procedures following primary surgery, where we could only estimate that the size of remaining squamous tissue was close to zero, relying on the judgement of the surgeon (indirect measurement). To lessen the biasing effect of our presumtion that indirectly measured cholesteatomas’ size was close to zero, we also ran the simulation with a starting size of 0.33 mm, 0.66 mm, and 1 mm. When comparing these methods, we found that cholesteatomas measured indirectly seemed to grow faster when a starting size of 0 was assumed, and had a wider range of values. As the starting size was increased through 0.33 mm and 0.66 mm and 1 mm, the difference in growth speed faded without a clinically significant change in detectability thresholds.

Although these indirect measurements are technically less precise than direct radiological follow up of the same cholesteatoma using MRI scans, it is of paramount importance to take these results into consideration, as indirect growth rate may better correspond to the typical clinical course of cholesteatoma cases in real life, where patients with cholesteatoma are getting operated on, then followed up, and when a residual or recurrent disease is detected during imaging, a revision surgery is performed. For us, the effect of different starting points in indirect growth sizes indicates that presuming an intraoperative residual cholesteatoma size closer to 1 mm might be more realistic.

Most cholesteatomas in this analysis were measured radiologically, apart from 5 cases of direct measurements, extracted from a study conducted considerably earlier [45]. To account for the heterogeneity caused by this, we also ran an analysis excluding these cases (Online Resource Fig. 6–7), which yielded results close to the original model (3 mm threshold at 27 months, 5 mm threshold at 46 months).

When analyzing basic demographic features, we found a slightly faster growth rate in males; however, age at surgery was not found to be a prognostic factor for growth rate. This latter finding was consistent with the results of a recent large retrospective cohort study, in which residual disease did not appear earlier in children than in adults [12]. Although pediatric cholesteatoma is traditionally thought to be more aggressive, this perception is mainly based on the primary extension upon diagnosis, the extended bony erosion, and observed biochemical properties compared to adults [10, 61, 62] rather than the speed of residual disease expansion.

We also examined whether the space in which residual cholesteatomas develop affects the rate of growth, which is closely related to the type of surgery. Theoretically, there may be a difference between an aerated mastoid space, a post-bony-obliteration mastoid bowl, and a cavity filled with autologous fat after subtotal petrosectomy [63]. Kemps et al. in a recent retrospective comparative study established that residual diseases were found earlier in the bony obliterated group compared to subtotal petrosectomy, although it was not indicated whether this difference was significant [64]. We performed a multivariate analysis comparing cholesteatoma growth rate in patients after tympanomastoidectomy with and without bony obliteration of the mastoid cavity, and in patients who had previously undergone subtotal petrosectomy. The results showed no difference between the groups. It should be noted that the number of cases was relatively low, with asymmetric distribution between the groups, the type of growth measurement was inhomogeneous, and the type of surgery was not randomized.

Several other aspects of residual cholesteatoma growth that would be of interest for further studies which our working group was unable to explore due to data scarcity. For example, it would be important to know whether there is a correlation between growth rate and the localization of the residual cholesteatoma (epitympanum, mesotympanum and mastoid cavity), presence of foreign material in the middle ear cavity such as prostheses or silicone sheets, and age at diagnosis of residual disease.

### Strengths

To our knowledge, this is the most comprehensive synthesis of the available data in the literature on the growth rate of residual cholesteatoma based exclusively on IPD. The main results of our study are based on reproducible and objective measurements obtained with non-EPI DWI MRI, the contemporary imaging modality for cholesteatoma follow-up and a simulation model based on the pooled data to estimate the growth rate across a large cohort.

### Limitations

Data were gathered from studies with a high risk of bias conducted under varying conditions, particularly with respect to surgery type, modality of follow-up, its timing, and the method of cholesteatoma size measurement. MRI protocols varied significantly, even within study populations, and most measurements were collected retrospectively, after diverse procedures. Little information was available on the initial extent of cholesteatomas, indications for certain types of procedures, and confounding factors. Cholesteatoma growth was simulated using an idealized shape; however, due to the complex anatomy of the middle ear, actual growth patterns are likely to be more intricate, which may have led to deviations in our results.

## Conclusion

Our simulation model based on individual participant data indicates that a 3 mm residual disease may first be radiologically detected 28–35 months postoperatively in 95% of patients, and a 5 mm residue might first be discovered at 47–58 months during surgery. Consequently, contrasting current clinical practice, delayed follow-up imaging in average-risk patients might reduce the rate of false negative screening results and increase cost-effectiveness. Rather than a new guideline defining an exact growth rate, our model, based on retrospective and heterogeneous data, aims to show patterns in cholesteatoma growth. This study is mainly intended to support future recommendations for ideal timing of follow-up imaging or second-look surgeries based on large-scale projects with less heterogeneity, individual participant data, and risk factor analysis for residual cholesteatoma growth.

## Supplementary Information

Below is the link to the electronic supplementary material.

Supplementary Material 1 (PDF 869 KB)

## Data Availability

The datasets used in this study can be found in the full-text articles included in the individual patient analysis, except for the data kindly provided by the working group at Université Paris Cité.
