# Supplementary material for: Modeling residual cholesteatoma growth rate: a meta-analysis of individual participant data
Source: Eur Arch Otorhinolaryngol. 2026 Jun 1;283(9):5609–20. doi: 10.1007/s00405-026-10326-5 (PMC13615138; doi:10.1007/s00405-026-10326-5)
Supplement: Supplementary file 1 [file 405_2026_10326_MOESM1_ESM.pdf]

Modeling residual cholesteatoma growth rate: a meta-analysis of individual patient data

Online Resource

Borbála Körmendy MD[1,2] (kormendy.klara@stud.semmelweis.hu, ORCID ID: 0000-0002-1534-2201), Judit Kálmán MD PhD[2] (kalmanj87@gmail.com, ORCID ID: 0000-0001-5515-7361), Kata Illés MD PhD[2] (i.kataaaa@gmail.com, ORCID ID: 0000-0002-2939-060X), Péter Fehérvári PhD[1,3] (fehervari.peter@univet.hu), Alexander Schulze Wenning MD[1] (a.schulzewenning@gmail.com), Rita Nagy MD PhD[1,4] (nagyrita003@gmail.com), Péter Hegyi Prof. MD PhD[1,5] (hegyi.peter@semmelweis.hu), Francois Simon Prof. MD PhD[6] (f.simon@aphp.fr), Tamás Horváth MD PhD[1, 2] (horvath.tamas@bajcsy.hu, ORCID ID:0000-0001-9617-8628)

[1]Centre for Translational Medicine, Semmelweis University, Budapest, Hungary

[2]Bajcsy-Zsilinkszky Hospital, Department of Otorhinolaryngology, Head and Neck Surgery, Budapest, Hungary

[3]University of Veterinary Medicine Budapest, Budapest, Hungary

[4]Heim Pál National Pediatric Institute, Budapest

[5]Institute of Pancreatic Diseases, Semmelweis University, Budapest, Hungary

[6]Université Paris Cité, AP-HP, Hôpital Necker-Enfants Malades, Paris, France

Corresponding author:

Tamás Horváth MD, PhD

Postal address: 1106 Budapest, Maglódi út 89-91.

E-mail address: horvath.tamas@bajcsy.hu

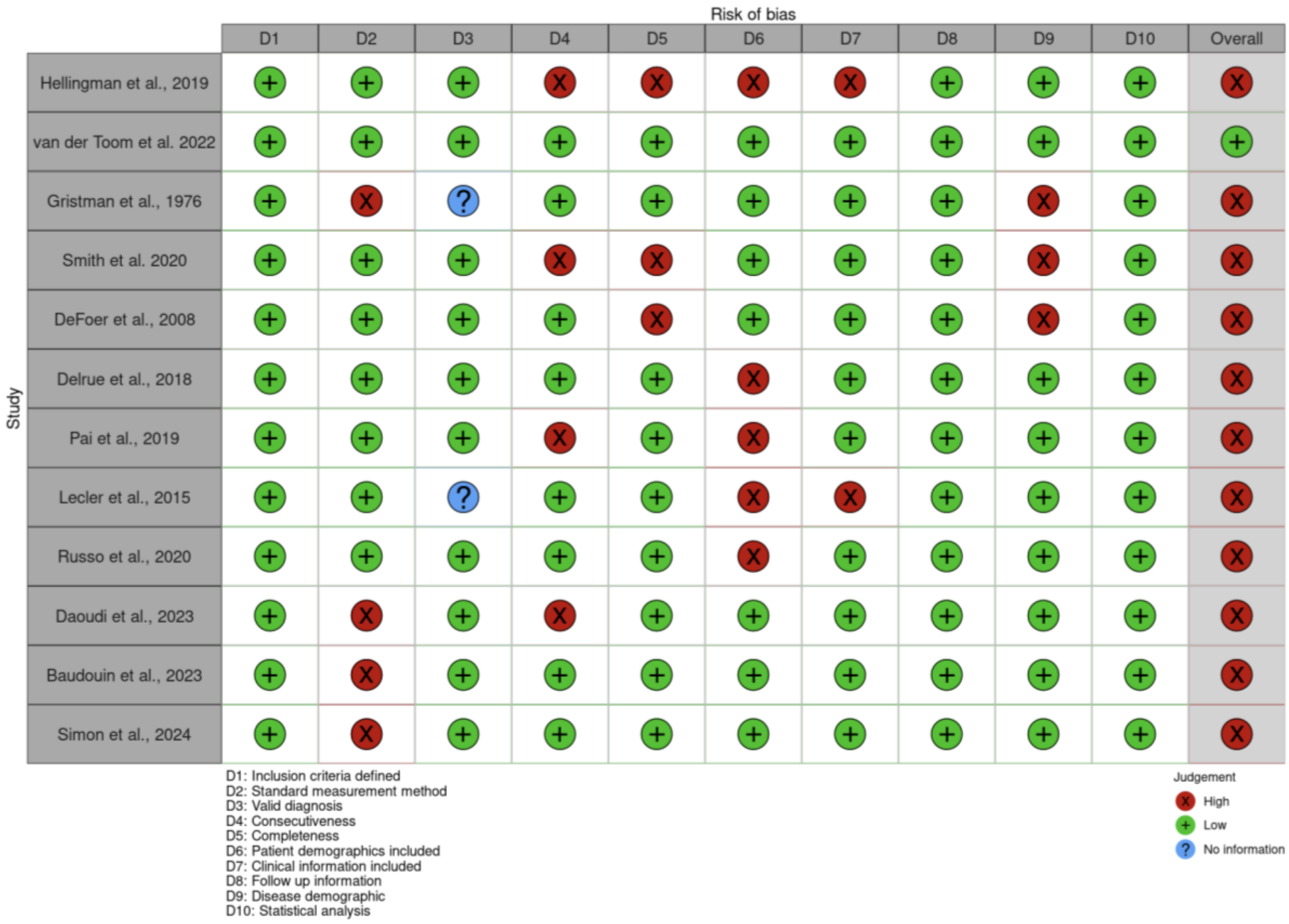

Online Resource Fig1 Risk of bias assessment accrding to the JBI checklist

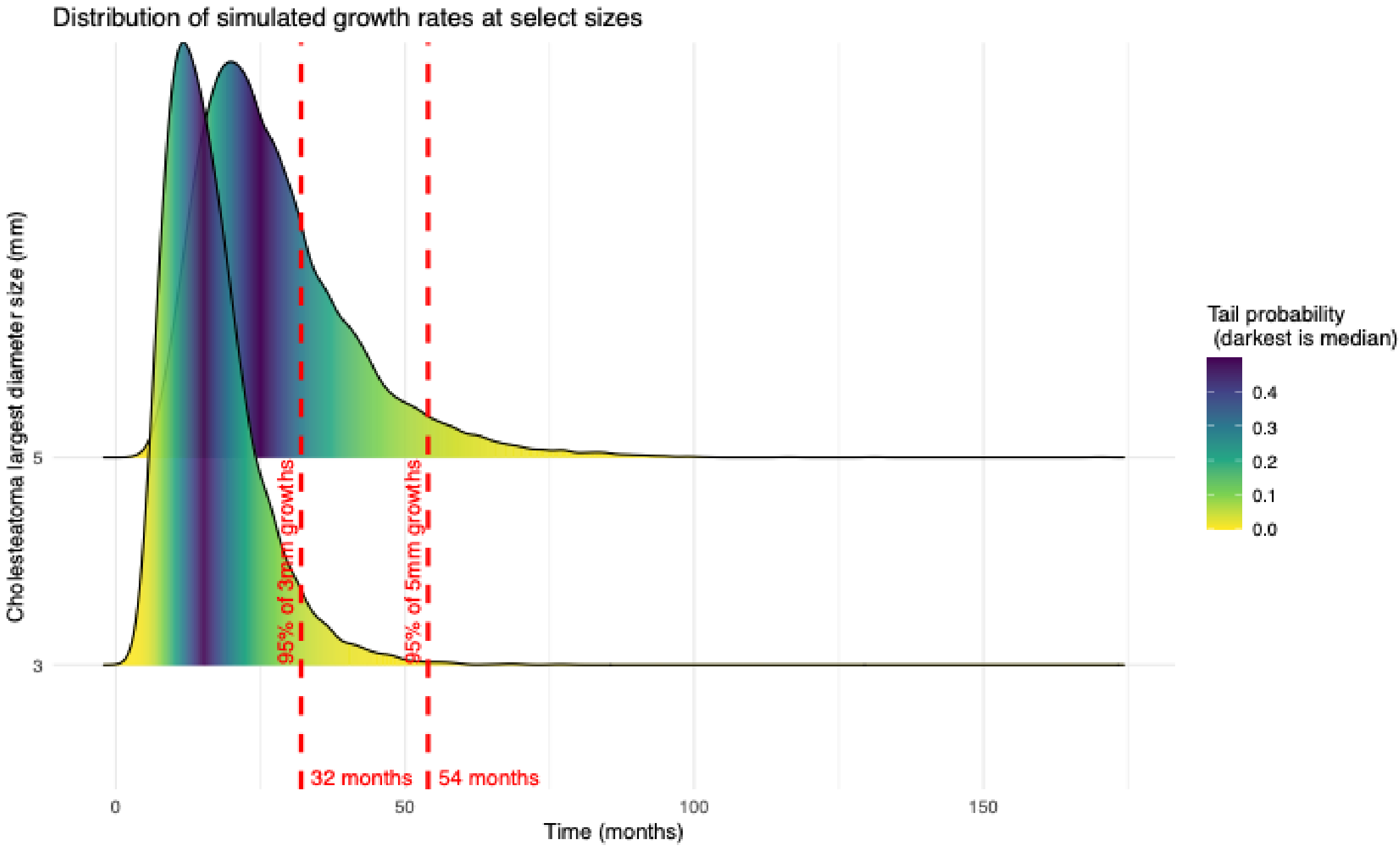

Online Resource Fig2

Ridge density plot of simulated time (months) required to reach 3 mm and 5 mm cholesteatoma growth targets, with the initial size of indirect cholesteatomas set to 0.33 mm. The figure shows the distribution of the likelihood of cholesteatomas to reach a size of 3 or 5 millimetres by a specific timepoint. Dashed red lines indicate 95th percentiles for each target.

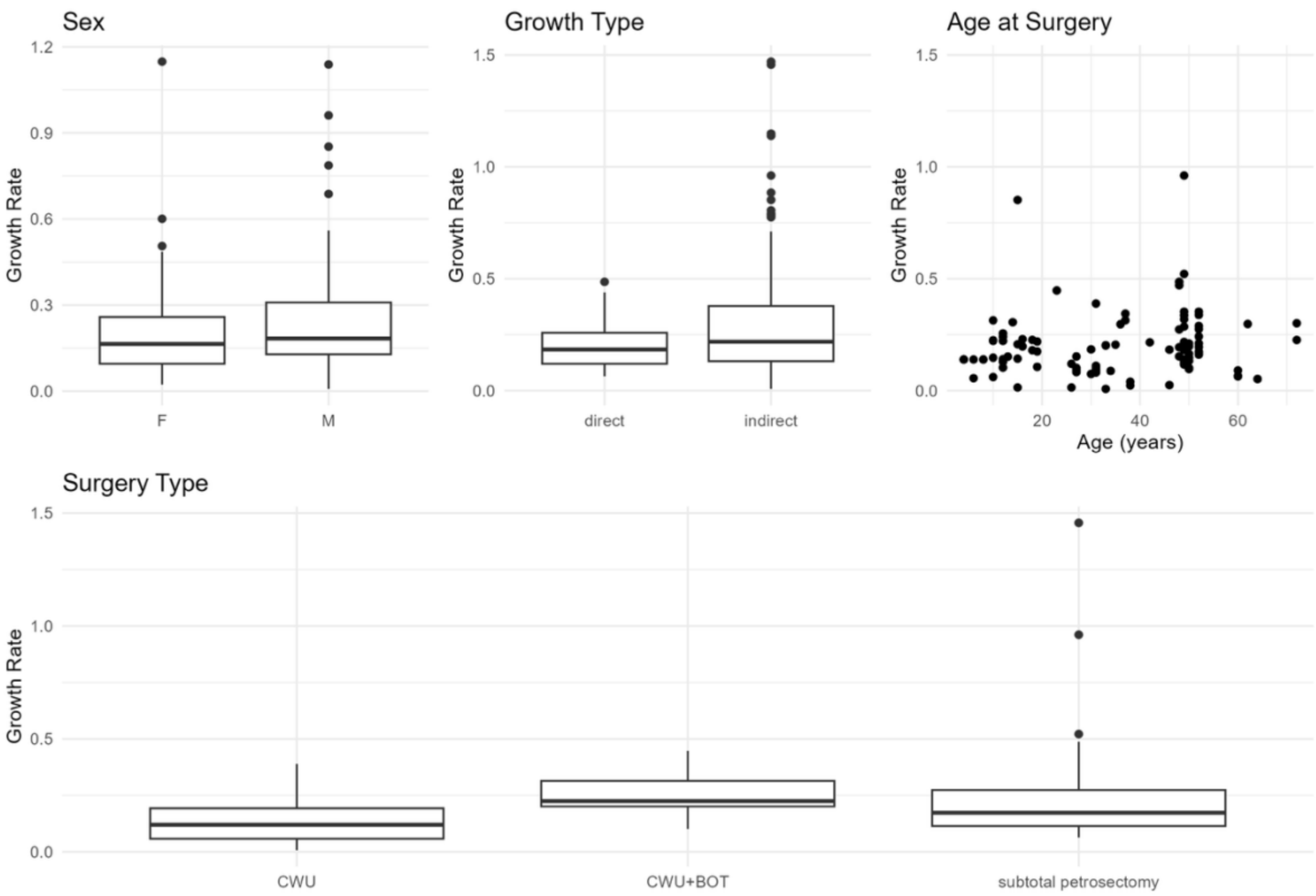

Online Resource Fig3

Summarization of observed relationships in the model with the initial size of indirect cholesteatomas set to 0.33 mm: top row includes sex, growth type, and age; bottom row shows effects of surgical type. mm: millimetre, M: male, F: female, CWU: canal wall up, CWU+BOT: canal wall up with bony obliteration

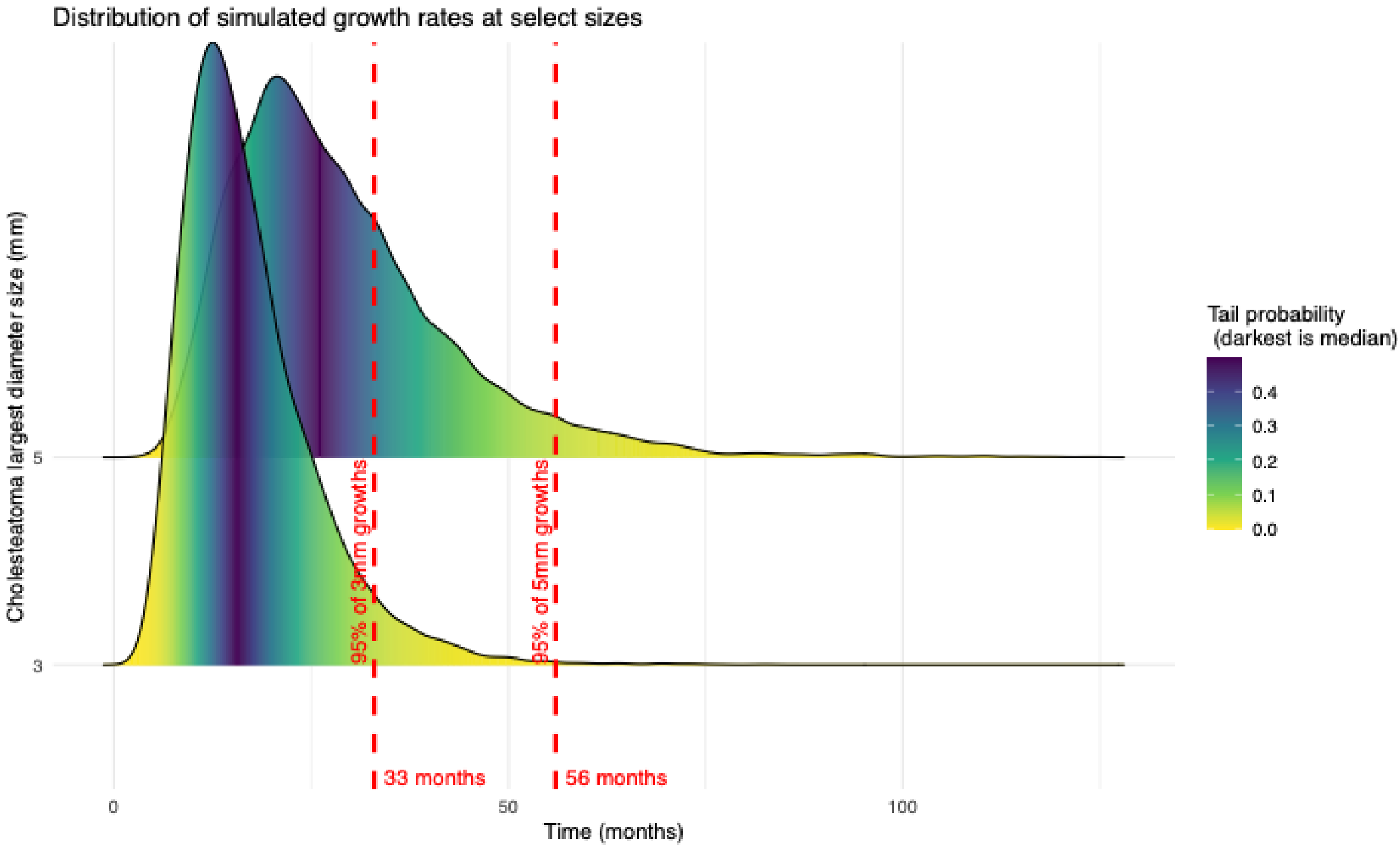

Online Resource Fig4

Ridge density plot of simulated time (months) required to reach 3 mm and 5 mm cholesteatoma growth targets, with the initial size of indirect cholesteatomas set to 0.66 mm. The figure shows the distribution of the likelihood of cholesteatomas to reach a size of 3 or 5 millimetres by a specific timepoint. Dashed red lines indicate 95th percentiles for each target.

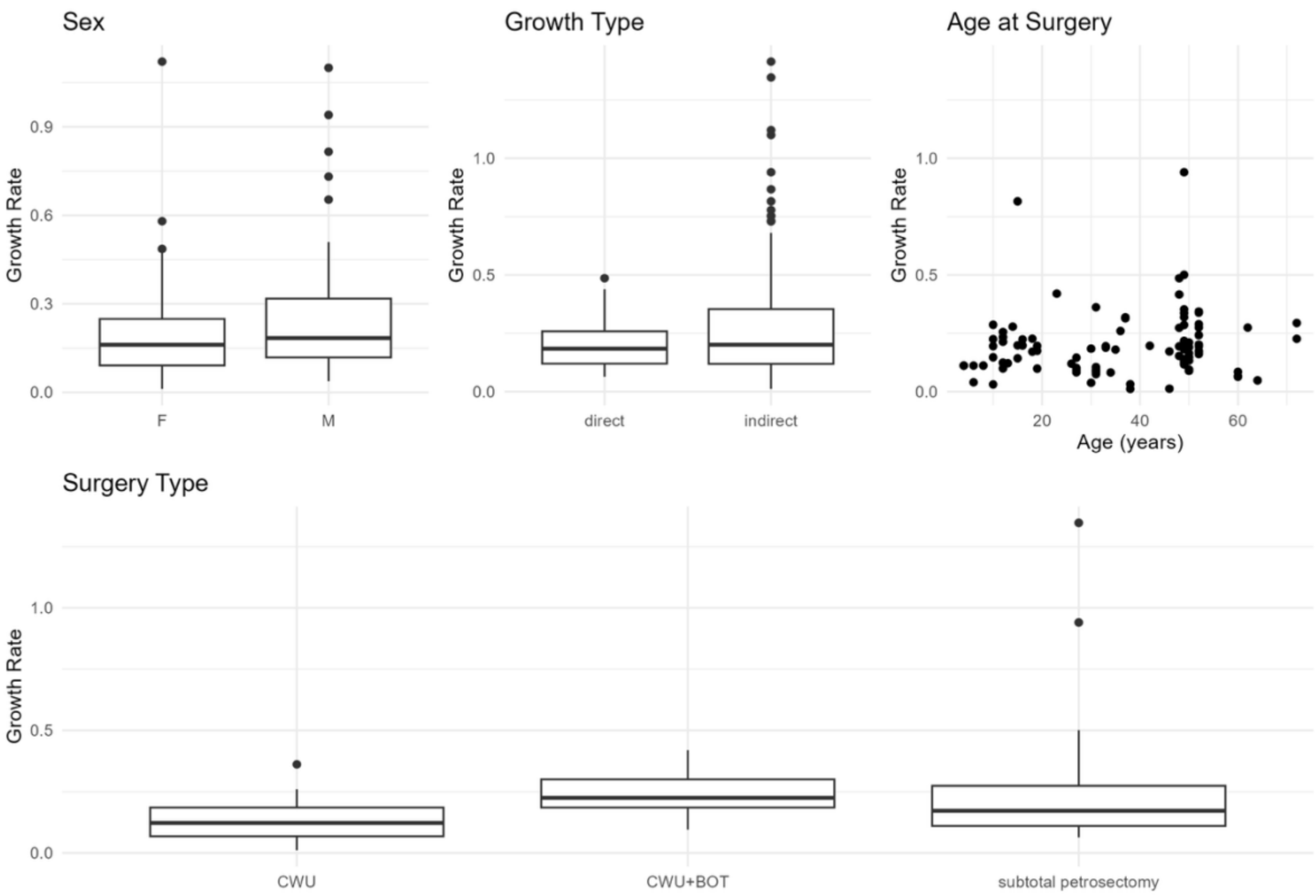

Online Resource Fig5

Summarization of observed relationships in the model with the initial size of indirect cholesteatomas set to 0.66 mm: top row includes sex, growth type, and age; bottom row shows effects of surgical type. mm: millimetre, M: male, F: female, CWU: canal wall up, CWU+BOT: canal wall up with bony obliteration

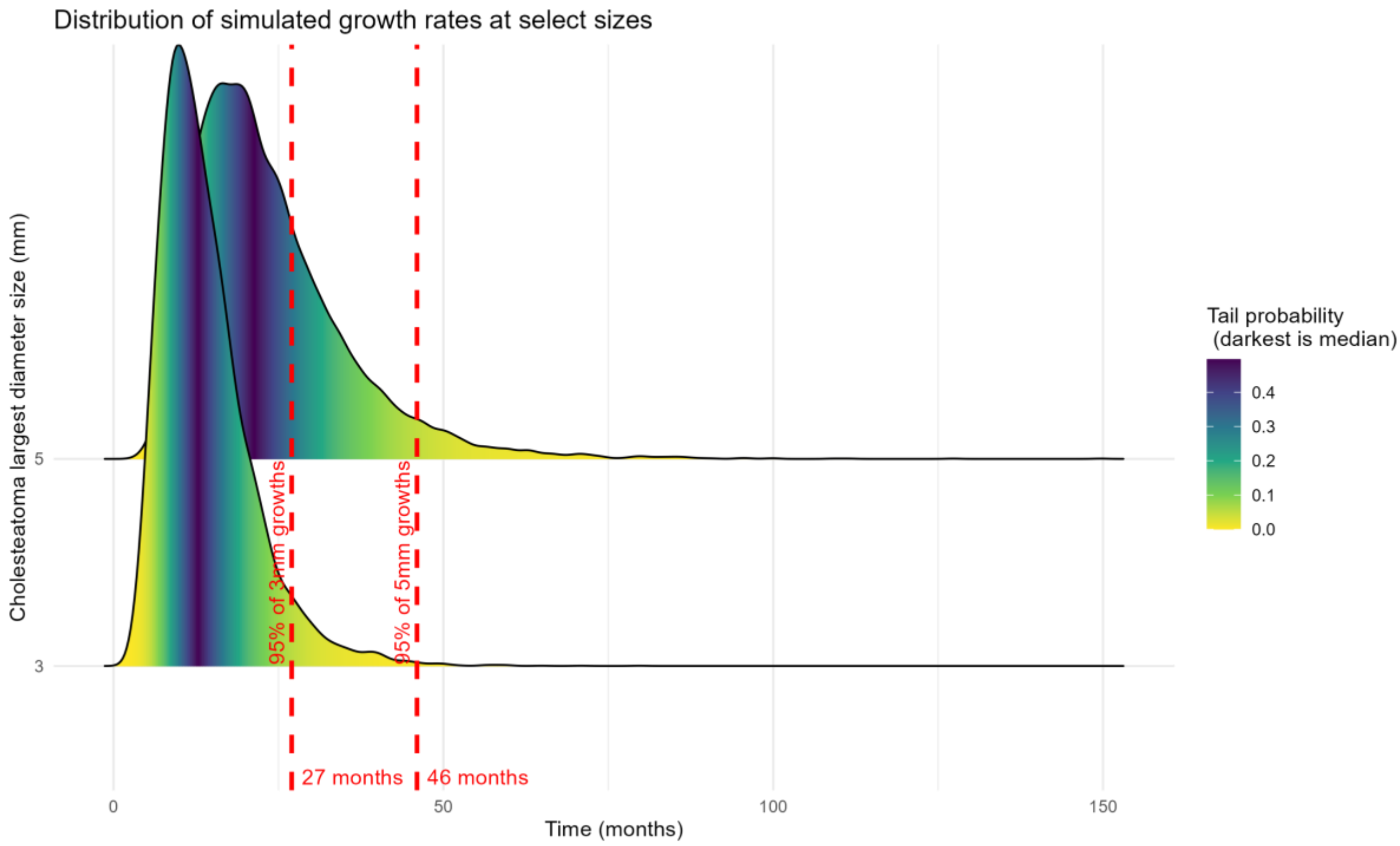

Online Resource Fig6

Ridge density plot of simulated time (months) required to reach 3 mm and 5 mm cholesteatoma growth targets, with only radiological measurements for indirect cholesteatoma growth. The figure shows the distribution of the likelihood of cholesteatomas to reach a size of 3 or 5 millimetres by a specific timepoint. Dashed red lines indicate 95th percentiles for each target.

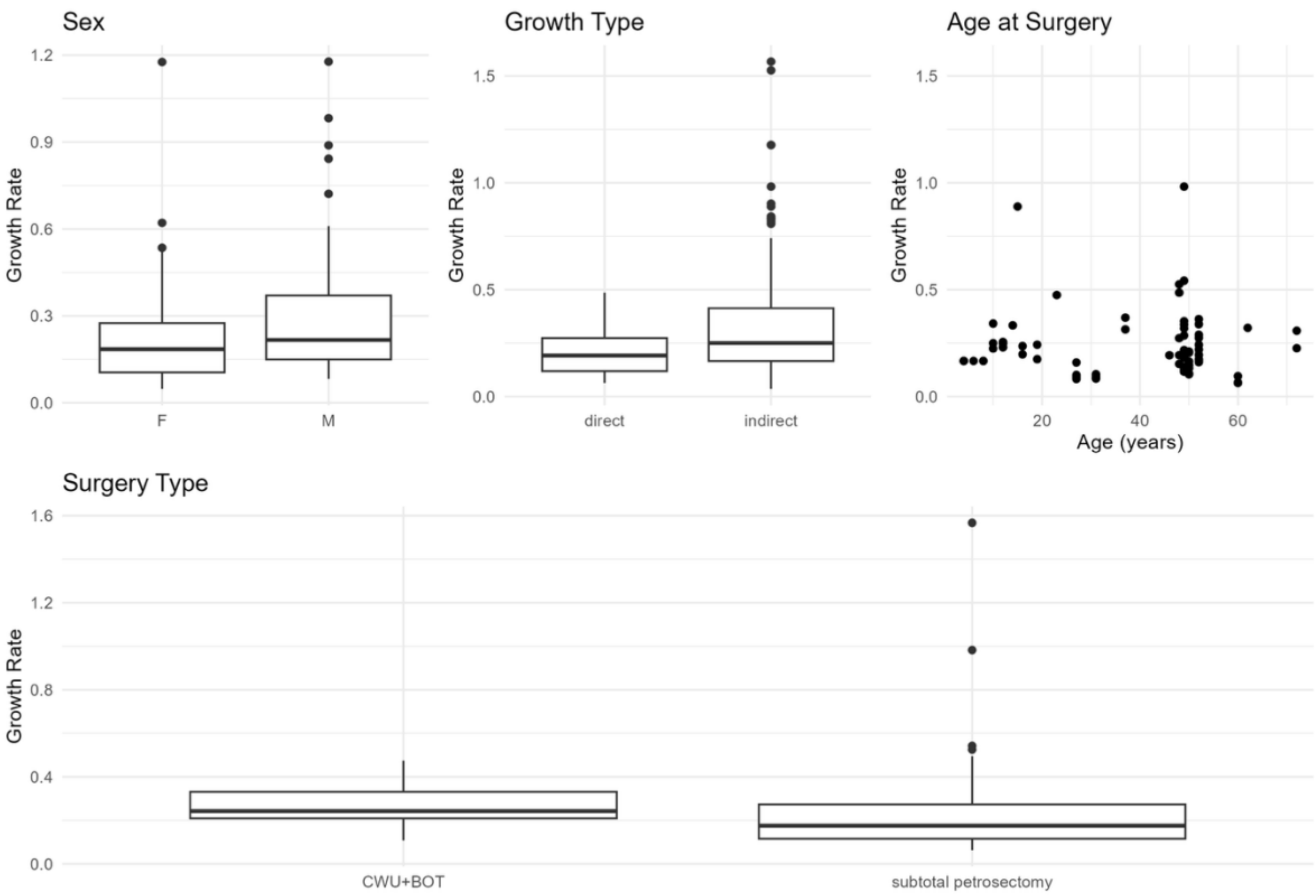

Online Resource Fig7

Summarization of observed relationships in the model with with only radiological measurements for indirect cholesteatoma growth: top row includes sex, growth type, and age; bottom row shows effects of surgical type. mm: millimetre, M: male, F: female, CWU: canal wall up, CWU+BOT: canal wall up with bony obliteration

Supplementary Material Table 1. Imaging protocols. T: tesla, non-EPI DWI MRI: non-echo planar diffusion weighted magnetic resonance imaging, HASTE: Half-Fourier Acquisition Single-shot Turbo spin Echo, TSE: Turbo Spin Echo, SE: Spin Echo, PROPELLER: Periodically Rotated Overlapping Parallel Lines with Enhanced Reconstruction, ADC: apparent diffusion coefficient, FOV: field of view

First author	Imaging modality	Slice thickness	FOV/matrix(mm)	Measurement method	Unit of mesurement
Baoudouin et al., 2023	1.5T or 3T non-EPI DWI MRI HASTE or PRROPELLER or TSE	NA	NA	NA	NA
Daoudi et al., 2023	1.5 T HASTE non-EPI DWI MRI, 1.5 T and 3 T PROPELLER non-EPI DWI MRI with ADC mapping	NA	NA	Largest diameter in the coronal plane	mm
Delrue et al. 2018	1.5 T non/EPI DWI MRI with ADC mapping	2 mm	NA	Coronal long axis and short axis diameter on T2 weighted images	mm, mm2
Hellingman et al. 2019	Varied MRI protocols (Siemens, Philips)	Varied MRI protocols	NA	Volumes based on manual segmentation	mm3, mm3/month

Lecler et al. 2015	1.5 T Non-EPI DWI, SE-T2, Dual SE T2, DWI b=0 and b=800s/mm2 and delayed contrast enhanced T1 W1	4,5 mm<br>1,7 mm<br>2,5 mm<br>2 mm	230/(256x168)<br>230/(336x212)<br>230/(176x100)<br>180/(205x204)	Not specified	mm
Pai et al. 2019	1.5 T non-EPI DWI MRI (HASTE) coronal planes, no ADC, b = 1000 s/mm2	3 mm	NA	Based on time interval between surgery and 1st positive scan or growth between positive scans, maximal coronal dimension	mm
Smith et al., 2024	non-EPI DWI MRI	NA	NA	Not measured	NA
Smith et al. 2020	non-EPI DWI MRI with ADC mapping	NA	NA	Maximal dimension in the axial and coronal planes	mm
van der Toom et al. 2022	1.5 T non-EPI DWI MRI, b=0 s/mm2 and b= 1000 s/mm2 with ADC mapping, coronal and axial TSE T2, coronal SE T1	2 mm	(220x220)/(134x192),<br>(150x200)/(192x256),<br>(150x200)/(144x256)	Largest diameter in the axial plane	mm

(Continued) Supplementary Material Table 1. Imaging protocols. T: tesla, non-EPI DWI MRI: non-echo planar diffusion weighted magnetic resonance imaging, HASTE: Half-Fourier Acquisition Single-shot Turbo spin Echo, TSE: Turbo Spin Echo, SE: Spin Echo, PROPELLER: Periodically Rotated Overlapping Parallel Lines with Enhanced Reconstruction, ADC: apparent diffusion coefficient, FOV: field of view

Supplementary Material Table 2. Non-radiological measurement methods. mm: millimeters

First author, year of publication	Mesuring method	Unit of mesurement
Gristwood et al., 1976	In-office and intraoperative measurement	mm
DeFoer et al., 2008	Intraoperative measurement	mm
